# The MUTYH base excision repair gene protects against inflammation-associated colorectal carcinogenesis

**DOI:** 10.18632/oncotarget.4284

**Published:** 2015-06-18

**Authors:** Francesca Grasso, Serena Di Meo, Gabriele De Luca, Luca Pasquini, Stefania Rossi, Monica Boirivant, Mauro Biffoni, Margherita Bignami, Emma Di Carlo

**Affiliations:** ^1^ Department of Environment and Primary Prevention, Istituto Superiore di Sanità, Rome, Italy; ^2^ Department of Science, University Roma Tre, Rome, Italy; ^3^ Ce.S.I. Biotech, Aging Research Center, “G. d'Annunzio” University Foundation, Chieti, Italy; ^4^ Pathological Anatomy and Molecular Medicine, Department of Medicine and Sciences of Aging, “G. d'Annunzio” University, Chieti, Italy; ^5^ Department of Hematology, Oncology and Molecular Medicine, Istituto Superiore di Sanità, Rome, Italy; ^6^ Department of Infectious Parasitic and Immuno-mediated Diseases, Istituto Superiore di Sanità, Rome, Italy

**Keywords:** MUTYH, azoxymethane, DSS, colorectal cancer, inflammation

## Abstract

MUTYH DNA glycosylase removes mismatched adenine opposite 7, 8-dihydro-8-oxoguanine (8-oxoG), which is the major mutagenic lesion induced by oxidative stress. Biallelic mutations in *MUTYH* are associated with MUTYH-Associated polyposis (MAP) and increased risk in colorectal cancer (CRC). We investigated cancer susceptibility associated with MUTYH inactivation in a mouse model of inflammation-dependent carcinogenesis induced by azoxymethane (AOM) and dextran sulphate (DSS). *Mutyh*^−/−^ mice were more sensitive than wild-type (WT) animals to AOM/DSS toxicity and accumulated DNA 8-oxoG in their gastrointestinal tract. AOM/DSS-induced colonic adenomas were significantly more numerous in *Mutyh*^−/−^ than in WT animals, and frequently showed a tubulo-villous feature along with high-grade dysplasia and larger size lesions. This condition resulted in a greater propensity to develop adenocarcinomas. The colon of untreated *Mutyh*^−/−^ mice expressed higher basal levels of pro-inflammatory cytokines GM-CSF and IFNγ, and treatment with AOM/DSS induced an early decrease in circulating CD4+ and CD8+ T lymphocytes and an increase in myeloid-derived suppressor cells (MDSCs). Adenomas from *Mutyh*^−/−^ mice had a greater infiltrate of Foxp3+ T regulatory cells, granulocytes, macrophages, MDSCs and strong expression of TGF-β-latency-associated peptide and IL6. Our findings indicate that MUTYH loss is associated with an increase in CRC risk, which involves immunosuppression and altered inflammatory response. We propose that the AOM/DSS initiation/promotion protocol in *Mutyh*^−/−^ mice provides a good model for MAP.

## INTRODUCTION

Base excision repair (BER) protects against oxidative DNA damage that arises as a by-product of normal cellular metabolism or from extrinsic sources. 7, 8-dihydro-8-oxoguanine (8-oxoG) is the most frequent mutagenic DNA lesion. 8-OxoG can mispair with adenine during DNA replication to generate G:C to T:A transversion mutations. Prevention of 8-oxoG–induced mutagenesis requires the cooperative action of two DNA glycosylases, the products of the *OGG1* and *MUTYH* genes. OGG1 removes 8-oxoG from 8-oxoG:C pairs and MUTYH scans the newly-synthesized daughter strand to locate and remove adenine mispaired with 8-oxoG [for reviews see 1–3]. *MUTYH* mutations are associated with colorectal cancer (CRC). Biallelic germline *MUTYH* mutations are responsible for MUTYH-associated polyposis (MAP), a recessively heritable colorectal polyposis that is linked to an increased risk of CRC [4–6]. Since biallelic *MUTYH* mutations confer a spontaneous mutator phenotype in human cell lines [7, 8] and in mice [9, 10], it is generally regarded that *MUTYH* inactivation in MAP patients drives genomic instability in colorectal epithelial cells thereby increasing CRC risk. Consistent with this view, CRC in MAP patients displays a distinctive molecular fingerprint of somatic G:C to T:A transversions in tumor suppressors and/or oncogenes including the adenomatous polyposis coli (*APC*) and *KRAS* genes [4–6]. The overall incidence of extra-intestinal malignancies was increased in MAP patients, with the tumour spectrum including cancer of the duodenum, ovary, bladder and skin [11].

Few studies have addressed the possible significance of *MUTYH* in sporadic CRC [12]. Although *MUTYH* mutations have been reported only rarely in sporadic CRC [13, 14], it remains possible that MUTYH inactivation has a larger role in sporadic cancer. Accumulation of oxidized DNA bases is associated with inflammatory bowel diseases (IBDs) and neoplasia in ulcerative colitis (UC) is accompanied by functional impairment of MUTYH. This impairment of BER has been proposed as an early event in UC-associated carcinogenesis [15].

Mice homozygous for inactive *Mutyh* show a limited cancer-proneness that is apparent only late in life (18 months) [16, 17]. Surprisingly, the colon is not the main target of transformation and *Mutyh* deficiency enhances small intestinal tumorigenesis spontaneously as well as in the genetic background of *Apc^Min/+^* mice [18]. In addition the major effect of treatment of *Mutyh*-null mice with KBrO_3_, a strong oxidizing agent, was an increase in tumors of the small intestine [17].

In a previous study we reported that MUTYH influences the inflammatory response in a mouse model of UC [19]. In this protocol animals were treated with dextran sulfate sodium (DSS) to disrupt the colonic epithelium and induce a robust inflammatory response. DSS-induced chronic inflammation is known to increase reactive oxygen and nitrogen species (RONS) that cause DNA oxidation [20, 21]. Our study revealed increased 8-oxoG levels in colon DNA of DSS-treated *Mutyh*^−/−^ mice [19]. This was not, however, linked to a measurable increase in cancer incidence. To examine further the CRC risk associated with MUTYH abrogation, we have used a two-stage, initiation-promotion carcinogenesis model. Inflammation-dependent carcinogenesis was induced by a combined administration of azoxymethane (AOM), followed by DSS-elicited promotion [22, 23]. Our findings demonstrate that MUTYH loss is indeed associated with an increased CRC risk. This increased risk may involve a specific, MUTYH-dependent modulation of the inflammatory response.

## RESULTS

### Single and combined exposure to AOM and DSS

**Figure 1:**
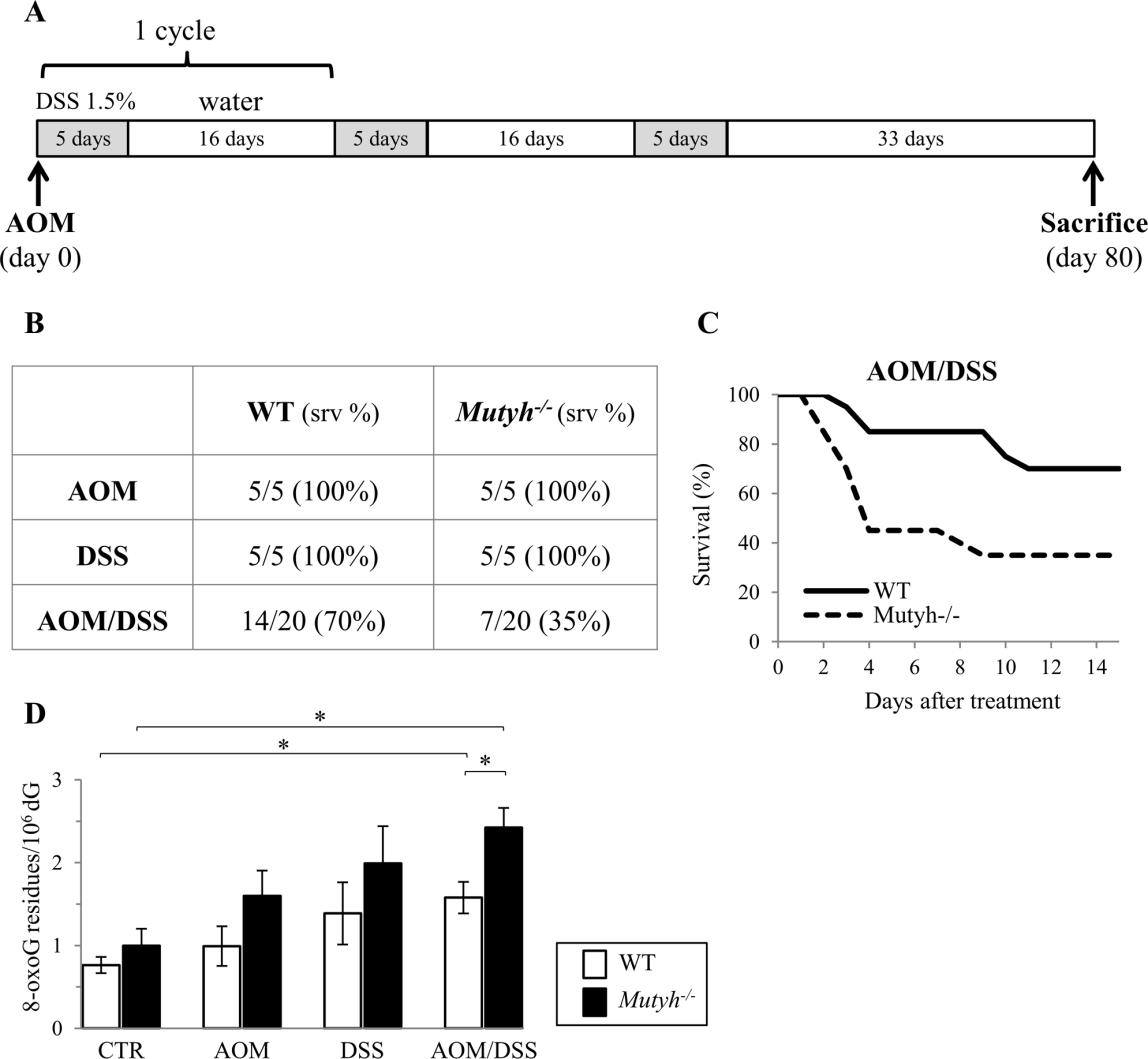
Survival and DNA 8-oxoG levels following single and/or combined AOM and DSS exposures **A.** Schematic representation of the treatment. **B.** Survival data of wild-type and *Mutyh*^−/−^ mice treated with AOM, DSS or the combined treatment as indicated in A). **C.** Kaplan–Meier survival curves of AOM/DSS-treated wild-type (solid line) and *Mutyh*^−/−^ (dotted line) mice. **D.** DNA 8-oxoG levels in the GI tract of wild-type (open bar) and *Mutyh*^−/−^ (full bar) mice untreated or exposed to AOM, DSS or combined treatment. Data are mean ± SE of 5–10 animals/genotype. *P ≤ 0.05 (Student's *t*-test).

To examine whether MUTYH acts as a suppressor of CRC, *Mutyh*^−/−^ and wild-type mice were treated with AOM followed by several cycles of DSS promotion. Twenty mice/genotype were treated with a single i.p. injection of 10 mg/kg AOM followed by exposure to 1.5% DSS in drinking water for three cycles (Figure 1A). Control groups (5 animals/group) included animals exposed to AOM only, DSS only, as well as untreated mice. All animals were sacrificed at 80 days from the beginning of the treatment. Under these experimental conditions, neither AOM nor DSS alone affected the survival of wild-type or *Mutyh*^−/−^ animals (Figure 1B). In contrast, combined AOM/DSS exposure had a synergistic effect and caused lethality in both genotypes. *Mutyh-* null mice were significantly more sensitive that wild-type to the combined treatment (70% vs 35% survival in wild-type and *Mutyh*^−/−^ mice respectively, *p* = 0.011 by Log-rank test) (Figure 1B). Toxicity occurred at early times with 13 *Mutyh*^−/−^ and 6 wild-type animals requiring early euthanasia between day 2 and day 10 (Figure 1C).

Although wild-type and *Mutyh*-null mice showed a tendency to accumulate DNA 8-oxoG in the gastrointestinal (GI) tract in all treatment conditions, a statistically significant increase was observed only in the AOM/DSS group (2.1-fold and 2.4-fold increases in wild-type and Mutyh-null mice, respectively) (Figure 1D). This synergistic effect was significantly more pronounced in *Mutyh*^−/−^ mice (1.5-fold increase over wild-type) (*p* = 0.012 by Student's *t*-test) (Figure 1D). In contrast, steady-state levels of DNA 8-oxoG were barely affected by MUTYH inactivation (Figure 1D and [10]).

Weight loss and shortening of colon length are parameters of inflammation in AOM/DSS mouse models [22, 23]. Weekly monitoring of the surviving animals did not reveal significant differences in body weight between the two genotypes and similarly colon length was unaffected by treatment or genotype (data not shown).

Taken together these results indicate that MUTYH protects against the toxicity of AOM/DSS and that this protection is associated with the removal of oxidative DNA damage.

### Early effects of AOM/DSS treatment

**Figure 2:**
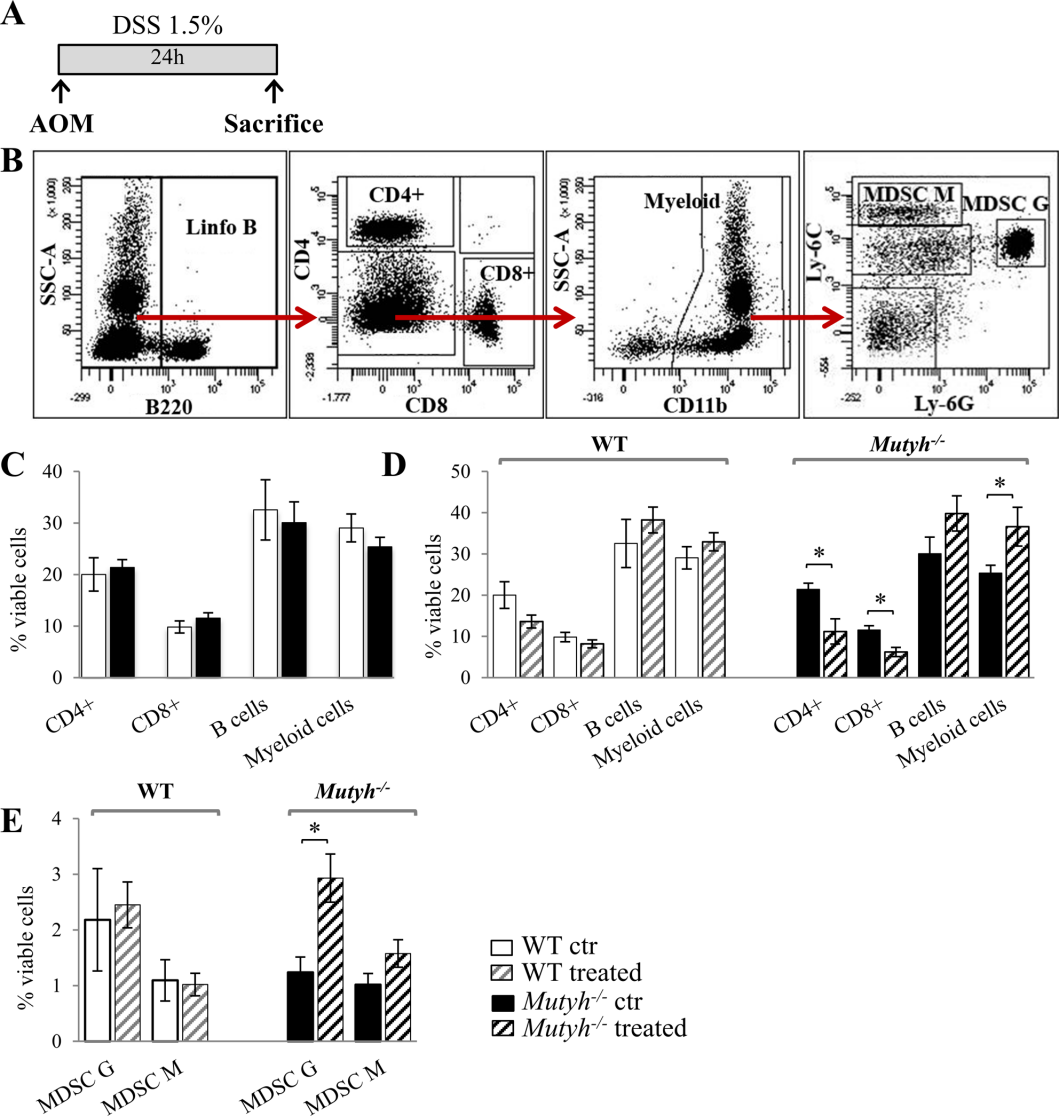
Analysis of leukocyte populations in the spleen or blood shortly after AOM/DSS exposures **A.** Schematic representation of the treatment. **B.** A representative image of FACS analysis. **C.** Leukocyte populations as percentages of viable cells in the blood of untreated wild-type (WT) (open bar) and *Mutyh*^−/−^ mice (full bar). **D.** Leukocyte populations as percentages of viable cells in the blood of untreated or AOM/DSS-treated mice. **E.** MDSCs populations (G: granulocytic, M: monocytic) as percentages of viable cells in the spleen of control or AOM/DSS treated mice. Untreated and treated wild-type mice (open and grey dashed bar, respectively); untreated and treated *Mutyh*^−/−^ mice (full and black dashed bar). Data are mean ± SE of 5 animals/genotype/condition. *P ≤ 0.05 (Student's *t*-test).

To examine the role of MUTYH in the early inflammatory events, AOM-treated mice were sacrificed after 24 h of DSS promotion (Figure 2A). Leukocyte populations (CD4+ and CD8+ T lymphocytes, B lymphocytes and myeloid cells) derived from spleen and blood were analysed by FACS. A representative image of these analyses is shown in Figure 2B. There were no striking differences in basal leukocyte populations either in the blood (Figure 2C) or in the spleen (data not shown) of wild-type and *Mutyh*^−/−^ mice. The leukocyte populations in the blood of *Mutyh*^−/−^ mice were, however, affected by AOM/DSS treatment with significant decreases in CD4+ and CD8+ T lymphocytes along with an increase in the myeloid population (Figure 2D). A more detailed analysis of the myeloid population revealed that AOM/DSS treatment increased both subpopulations of myeloid-derived granulocytic and monocytic suppressor cells (MDSCs). The change in granulocytic MDSCs in KO mice reached statistical significance whereas the levels remained unchanged in wild-type mice (Figure 2E).

We conclude that MUTYH prevents an early increase in MDSCs induced by AOM/DSS exposure. Consistent with this observation, we noted that after 80 days of AOM/DSS treatment the spleen weights were significantly increased in KO but not in wild-type mice (Figure 1 supplementary). Histological analyses of splenomegaly in *Mutyh*^−/−^ mice revealed a wider extra-lymphoid compartment compatible with an expansion of the myeloid fraction.

**Figure 3:**
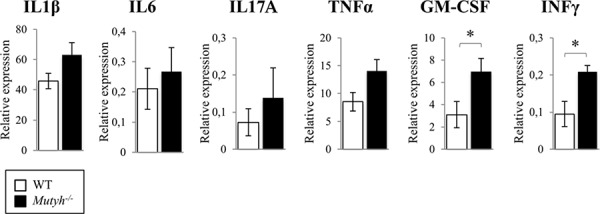
Analysis of cytokines expression in the colon Cytokines basal levels in the colon of wild-type (open bar) and *Mutyh*^−/−^ (full bar) were measured with Bio-Plex Pro™ Mouse Cytokine 8-plex Assay. Proteins levels are expressed as concentration (pg/ml)/mg of total proteins. Data are mean ± SE of 4–5 animals/genotype. *P ≤ 0.05 (Student's *t*-test).

We previously reported that *Mutyh*^−/−^ differ from wild-type mice in the expression of certain inflammation-related cytokines in the GI tract [19]. By means of a multiplex cytokine fluorescent bead-based immunoassay, that allows the simultaneous detection of several cytokines, we evidenced in the colon of untreated *Mutyh*^−/−^ mice, a trend towards higher basal levels of IL1β, IL6, IL17A, GM-CSF, TNFα, in comparison with colon from wild type animals, whereas differences reached statistical significance for GM-CSF and INFγ (Figure 3). The levels of these pro-inflammatory cytokines were decreased by AOM/DSS (one cycle) treatment. This reduction was more pronounced in *Mutyh*^−/−^ than in wild-type animals (Figure 2 Supplementary).

The increased basal levels of pro-inflammatory cytokines in the colon of *Mutyh*^−/−^ mice indicate that MUTYH inactivation is associated with an inflammation-prone phenotype. In contrast, MUTYH inactivation does not appear to impair the early response to AOM/DSS treatment.

### Colonic adenomas and adenocarcinomas induced by AOM/DSS treatment

**Figure 4:**
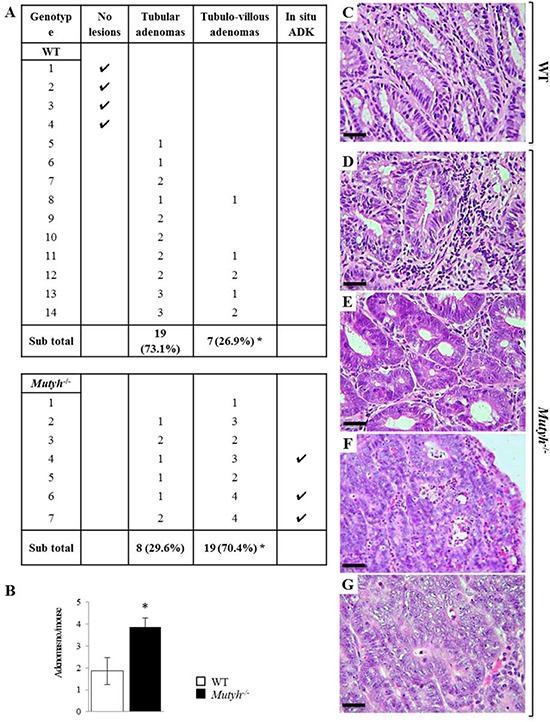
Morphologic features of colonic tumors following AOM/DSS treatment **A.** Histologic alterations developed in the colon of wild-type and *Mutyh*^−/−^ mice following AOM/DSS treatment. *P = 0.016 (Fisher's exact test). **B.** Average number of adenomas/mouse in wild-type (open bar) and *Mutyh*^−/−^ (full bar). Data are mean ± SE of 7–14 animals/genotype. *P ≤ 0.05 (Student's *t*-test). Representative images of H&E stained sections of tubular adenomas with a mild/moderate dysplasia in wild-type (C) and *Mutyh*^−/−^ (D) mice. Adenomas with severe dysplasia were more frequent in *Mutyh*^−/−^ mice (E) which may also develop adenocarcinomas (F, G). Adenocarcinoma (ADK). Magnification: C, D, E, G, X400; F, X200. Scale bars: C, D, E, G, 30 μm; F, 50 μm.

Following exposure to DSS/AOM, 10/14 wild-type mice (71%), and 7/7 *Mutyh*^−/−^ mice (100%) developed tubular or tubulo-villous colonic adenomas (Figure 4A and 4C–4F). The tubulo-villous feature was more frequent among adenomas in *Mutyh*^−/−^ than in wild-type mice (Fisher's exact test, *p* = 0.016) (Figure 4A and 4E). In addition *Mutyh*^−/−^ adenomas were larger and exhibited higher grades of dysplasia. These features are consistent with enhanced carcinogenesis (24). Indeed, three out of seven *Mutyh*^−/−^ mice, developed adenocarcinomas (Figure 4A and 4F, 4G), while none were observed in wild-type mice.

Treatment with AOM alone gave rise to a slight inflammatory reaction and the development of hyperplastic polyps. These were identified in 1/5 wild-type and 3/5 *Mutyh*^−/−^ mice, which developed respectively 1, and 2 to 5 lesions. No remarkable genotype-dependent differences were observed in mice treated with DSS alone. This treatment provoked an inflammatory infiltrate of granulocytes, lymphocytes and plasma cells in the colonic mucosa of both wild-type and *Mutyh*^−/−^ mice (data not shown).

**Figure 5:**
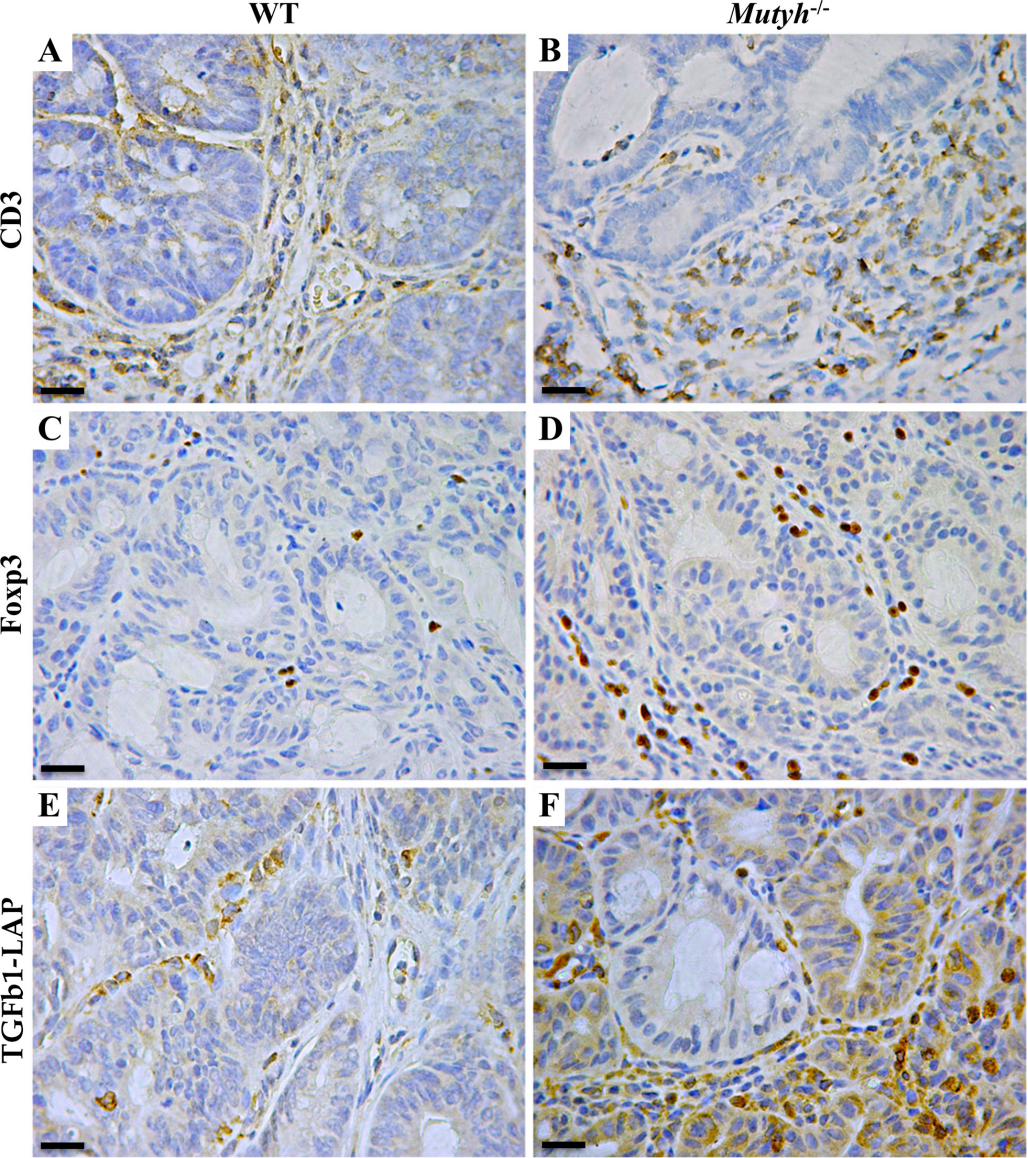
Lymphocyte infiltrate, Foxp3 and TGFβ1-LAP expression in adenomas developed in AOM/DSS-treated wild-type and *Mutyh*^−/−^ mice **A–F.** Representative images of IHC performed on paraffin-embedded sections, showing CD3^+^ T lymphocyte infiltrate in the stromal component of adenomas developed in wild-type (A), and *Mutyh*^−/−^ (B) mice. Foxp3^+^ lymphocytes were barely detected inside adenomas from wild-type mice (C), while they were more represented inside adenomas from *Mutyh*^−/−^ mice (D). TGFβ1-LAP expression in wild-type (E), and *Mutyh*^−/−^ (F) mice. Magnification: A–F, X400. Scale bars: A–F, 30 μm.

Immunohistochemical analyses of the AOM/DSS-induced adenomas revealed a genotype-independent infiltrate of CD3^+^ T lymphocytes in the stromal compartment (Table 1, and Figure 5A, 5B). B220^+^ lymphocytes were almost undetectable and were mainly confined to the lymphoid follicles in the colonic *lamina propria* or between this and the submucosa. As already reported [19], lymphoid follicles were larger and more numerous in *Mutyh*^−/−^ than in wild-type mice.

**Table 1:** Immunohistochemical analyses of adenomas induced by AOM/DSS

	WT	*Mutyh*^−/−^
***Immune cells***		
CD3	9.0 ± 3.5	14.0 ± 5.2
Foxp3	4.0 ± 1.5	11.0 ± 3.8^*^
Granulocytes	2.0 ± 1.5	11.3 ± 4.0^*^
Macrophages	13.0 ± 4.5	23.5 ± 5.0^*^
MDSCs	5.5 ± 2.2	15.0 ± 4.5^*^
***Cytokines***^#^		
IL6	±	+ +
TGFβ1-LAP	±	+ +
IFNγ	−	−
TNFα	±	±

Counts of immune cells and assessment of cytokines were performed at X400 in a 0.180 mm^2^ field. At least 3 samples (three sections/sample), and 6–8 (depending on the tumor width) randomly chosen fields/section were evaluated. Results are expressed as mean ± SD of CD3 (lymphocytes), Foxp3 (regulatory cells), GR-1 (granulocytes), CD11b/CD18 (macrophages) or CD11b/GR-1 (MDSCs) positive cells per field evaluated on paraffin embedded or cryostat (macrophages) sections by immunohistochemistry.

# The expression of cytokines was defined as absent (─); scarce (±); distinct (+) or strong (++) on paraffin embedded sections stained with the corresponding Ab.

* Values significantly different (*P* < 0.05) from corresponding values in tumors developed in WT mice.

**Figure 6:**
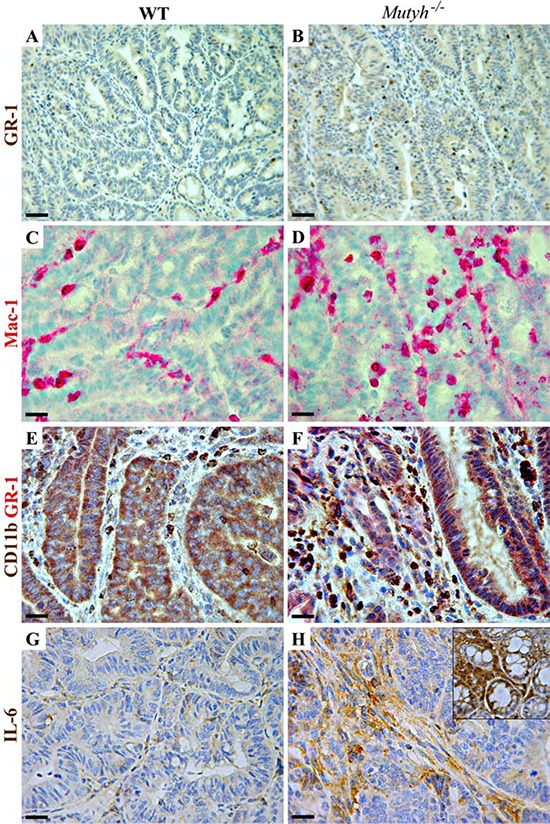
Myeloid cell infiltrate and IL6 expression in adenomas developed in AOM/DSS-treated wild-type and *Mutyh*^−/−^ mice **A–H.** Representative images of IHC performed on paraffin-embedded or frozen (C, D) sections of adenomatous lesions developed in wild-type (left column) or *Mutyh*^−/−^ (right column) mice after AOM/DSS treatment. A–B) Granulocytes (GR-1^+^) staining in wild-type (A) and in *Mutyh*^−/−^ mice (B) C–D) Macrophages (CD11b^+^/CD18^+^: Mac-1) staining in wild-type (C) and in *Mutyh*^−/−^ mice (D) E–F) Double immunohistochemistry with anti-GR-1 (red), and anti-CD11b (brown) Abs, resulting in double stained MDSCs (brick red staining) inside adenomas from wild-type (E) and *Mutyh*^−/−^ mice (F) G–H) Expression of IL6 in adenomas from wild-type (G) and *Mutyh*^−/−^ mice (H), and within the nearby normal colonic crypts (H inset). Magnification: A and B, X200; C–H, X400. Scale bars: A and B, 50 μm; C–H, 30 μm.

Compared to those in wild-type mice, adenomas from *Mutyh*^−/−^ mice showed a distinct infiltrate of Foxp3^+^ regulatory T cells (Table 1, Figure 5C, 5D), along with a concomitant strong expression of TGF-β1 latency-associated peptide (LAP) (Table 1 and Figure 5E, 5F) visible both in infiltrating cells and in the dysplastic epithelium (Figure 5F). Moreover, granulocytes (GR1+), macrophages (Mac1+) and MDSCs (CD11b+/GR1+) in association with a high level of IL6 expression (Table 1, Figure 6A–6H) were also significantly (*P* < 0.05) more represented in *Mutyh*^−/−^ adenomas. In contrast, TNFα was barely expressed and IFNγ was undetectable (Table 1).

We conclude that MUTYH loss affects both the number and the severity of neoplastic lesions induced by this treatment. In addition this repair gene modulated, both qualitatively and quantitatively, the immune response associated with these lesions.

## DISCUSSION

MUTYH inactivation in mice is associated with small intestinal tumour and/or lymphomas, either spontaneous or induced by KBrO_3_ exposure [17]. In addition, similarly to inactivation of other mismatch repair (MMR) genes (*Mlh1*^−/−^, *Msh2*^−/−^, *Msh6*^−/−^, *MBD4*^−/−^) [25–28], the small intestine is the preferential target for accelerated carcinogenesis in *Mutyh*/*Apc^Min/+^* crosses [18]. The striking specificity of the small intestine as major target site for tumorigenesis in rodents has been recently explained by the larger number of stem cells divisions occurring in mouse small intestine than in the large one, with the reverse occurring in humans [29]. Here we report that the colon becomes the target organ for Mutyh-dependent cancer when animals are exposed to an initiation-promotion protocol of inflammation-associated colorectal carcinogenesis. Since no cancer was observed in *Mutyh*^−/−^ mice exposed to several cycles of DSS alone [19], we conclude that the initiation step provided by AOM is required to reveal the *Mutyh*^−/−^ dependent CRC-prone phenotype.

The increased cancer susceptibility of *Mutyh*^−/−^ mice is accompanied by hypersensitivity to the lethality of AOM/DSS treatment. This phenotype is shared with mice defective in other DNA repair enzymes, i.e. O^6^-methylguanine DNA methyltransferase (MGMT) [30, 31], alkyladenine DNA glycosylase (AAG) and the ALKBH2/ALBH3 demethylases [21, 32]. These enzymes are major repair systems for mutagenic/cytotoxic DNA lesions induced by AOM. In contrast the acknowledged repair function of MUTYH is restricted to removal of a pre-mutagenic lesion – an undamaged adenine opposite 8-oxoG. The increased levels of DNA 8-oxoG identified in the GI tract of AOM/DSS-treated *Mutyh-* defective mice indicate that MUTYH is required for the efficient removal of this DNA lesion. Our data support a more complex role of MUTYH in mediating toxicity induced by DNA damaging agents [33, 34]. Modulation of MUTYH expression has been shown to be among the genomic predictors of cellular sensitivity to alkylation damage induced by *N*-methyl-*N’*-nitro-*N*-nitrosoguanidine [35]. Whether MUTYH influences alkylation sensitivity *via* its interaction with the MMR machinery remains to be determined. Indeed, depending on the oxidant and/or the type of damage to macromolecules, the absence of MUTYH can sensitize cells to DNA damaging agents or be beneficial for cell survival. We previously showed a surprising resistance of *Mutyh*^−/−^ mice to killing induced by combined Azathioprine (AZA) and UVA radiation chronic exposures [36]. These treatments lead to increased levels of skin DNA 8-oxoG and squamous cell skin carcinomas that arose only in *Mutyh*^−/−^ mice [36]. Thus, a cancer-prone phenotype is always associated with MUTYH loss, independently from the contrasting toxicities induced by AOM/DSS or AZA/UVA exposures.

Our findings indicate that a MUTYH-dependent modulation of the inflammatory response is associated with carcinogenesis. The inflammatory response is triggered and maintained by an intricate network of immune cells and molecules. Any disruption of this delicate balance may cause a switch from a normal microenvironment to one that supports tumour growth. In this regard the increased frequency of splenic MDSCs that we observed as an early event in AOM/DSS treated *Mutyh*^−/−^ animals suggests that a MUTYH defect compromises the maturation of myeloid-derived progenitors of antigen presenting cells. Increased MDSC levels are indeed associated with cancer in both mice and humans, where they might contribute to the immune evasion of transformed cells [37]. In addition, a MUTYH defect leads to an increased expression of certain pro-inflammatory cytokines. Taken together, these findings indicate that MUTYH inactivation modifies the baseline protective immunity as well as the immune response to external stimuli.

During colitis-associated cancer, tumor progression is driven essentially by an immune suppressive milieu that is more the result of deregulated immune signaling than physical damage to the colon. Our findings indicate that MUTYH loss affects this immune signaling. Adenomas from *Mutyh*^−/−^ animals display distinct features consistent with immunosuppression. Thus, whereas adenomas from wild-type and KO mice are similarly infiltrated by CD3+ T lymphocytes, *Mutyh*^−/−^ adenomas contain significantly more regulatory T cells (Tregs, Foxp3+). These cells have been shown to support colitis-associated cancer development by preventing the anti-tumor immune responses *via* inhibition of tumor-specific CD4+ cells and cytotoxic T lymphocytes [38]. Adenomas occurring in *Mutyh*^−/−^ mice show strong expression of TGF-β-LAP in comparison to wild-type mice, indicating an activated status of the Tregs cells [39]. This might occur *via* switching of tumor infiltrating CD4+ T cells to Foxp3+ Tregs by the high TGF-β level present in the immunosuppressive environment of the tumor [40, 41]. In addition Tregs recruitment can derive from secretion of Tregs-attractive chemokines by tumor-infiltrating macrophages [42]. Macrophages (as well as neutrophils) are indeed more represented in *Mutyh*^−/−^ adenomas suggesting that these cells might suppress antitumor immunity and be hijacked by tumor cells to support invasion [43, 43].

Another population of regulatory immune cells increased in the tumor environment of *Mutyh*^−/−^ mice are MDSCs. These cells can be generated in the bone marrow in response to factors such as IL-6, GM-CSF, IL-1β, TNFα and have been shown to contribute to cancer immune evasion by suppressing T cell functions and promoting activation and expansion of Foxp3+ Tregs cells [45, 46]. Thus MDSCs together with Tregs play a pivotal role in the creation and maintenance of an immunologically permissive tumor milieu. To complete the picture of a more severe and aggressive phenotype of adenomas developed in *Mutyh*^−/−^ mice, increased expression of IL6 was observed both in the tumor and within the nearby normal colonic crypts. The signal transduction induced by binding of this cytokine to its receptor leads to the transcription of several genes involved in tumor cell survival, angiogenesis, metastasis, inflammation [47]. Moreover an increased expression of IL6 has been associated with an unfavorable prognosis in patients with various types of cancer including sporadic and colitis-associated CRC [48, 49].

There are parallels between the rodent model for MUTYH inactivation and humans. These include a moderate mutator phenotype (7–10) and a relatively late age of tumor onset [50]. Although there is limited information on the natural history of adenoma and carcinoma development in MAP, some observations suggest that the malignant transformation onset and the progression of the disease might be accelerated in these patients [50]. Thus in MAP the risk of CRC appears not to be associated with the number of adenomas in the colorectum and a substantial proportion of patients developed CRC within the first decade after primary diagnosis of polyposis or a primary CRC [50]. Here we report an increased and earlier onset of malignant lesions in the colon of *Mutyh*^−/−^ mice which is associated with an aberrant inflammatory response following AOM/DSS treatment. By extrapolating our results to humans it is tempting to speculate that exposures to colon-specific carcinogens might contribute to increase the cancer risk in MAP patients.

## MATERIALS AND METHODS

### Animals and AOM and DSS treatment

A colony of *Mutyh*^−/−^ and littermate wild-type mice was maintained in the Laboratory Animal Services of Istituto Superiore di Sanità. All studies were conducted in accordance with the principles and procedures outlined in the EU (European Community Guidelines for Animal Care, DL 116/92, application of the European Communities Council Directive, 86/609/EEC), FELASA, and ARRIVE guidelines. The experimental protocol was approved by the Italian Ministry of Health. The animals were kept under standardized temperature, humidity, and lighting conditions, and had free access to water and food. All efforts were made to reduce the number of animals used and to minimize their suffering.

For colon carcinogenesis protocol, 8–10 week-old mice (20 mice/genotype) were given 10 mg/kg Azoxymethane (AOM) (Sigma Chemical Co., St. Louis, MO, USA) i.p. (day 0) and 1.5% DSS (MW:36, 000–50, 000, ICN Pharmaceuticals, Costa Mesa, CA, USA) in drinking water for 3 cycles (1 cycle: 5 days DSS + 16 days tap water). Mice were sacrificed 80 days after injection (cervical dislocation). The control groups (5 mice/genotype/group) were: mice given only AOM and ordinary tap water during the whole period, only DSS without injection and untreated mice. Body weights were recorded weekly. At the end of treatment spleen weight and colon length were measured. For histology and IHC, colon and spleen were fixed in 4% formaldehyde or frozen in Killik cryostat embedding medium (Bio Optica Milano S.p.A., Milano, Italy). For DNA isolation, small intestine sections were minced and snap frozen in liquid nitrogen within a few minutes after euthanasia.

For leucocytes population analyses 8–10 week-old mice (5 mice/genotype) were given 10 mg/kg AOM i.p. and 1.5% DSS in drinking water for 24 h and then were sacrificed with cervical dislocation. Spleen was removed and a blood sample was collected by cardiac puncture for FACS analysis.

For cytokines measurements 8–10 week-old mice (7–8 mice/genotype) were given 10 mg/kg AOM i.p. and 1.5% DSS in drinking water for 1 cycle and then were sacrificed with cervical dislocation; whole colon was minced and snap frozen in liquid nitrogen within a few minutes after euthanasia for proteins extraction.

### Histopathological and IHC analyses

Colon from the ileocecal valve to the anus was removed, washed in ice-cold saline, fixed in 10% neutral buffered formalin, embedded in paraffin, sectioned at 4 μm and stained with hematoxylin and eosin (H&E). Single and double IHC were done on paraffin-embedded or frozen sections, depending on the antibody (Ab). For IHC on frozen sections, large intestines were embedded in optimum cutting temperature compound (Miles), snap-frozen in liquid nitrogen and stored at −80°C. Then, frozen samples were sectioned, air dried overnight, fixed with acetone, and immunostained with anti-CD11b/CD18 (M1/70.5; Sera Lab, Crawley Down, UK) and anti-IFNγ (clone XMG1.2, BD PharMingen, San Diego, CA, USA) Abs. After washing, sections were overlaid with biotinylated rabbit anti-rat IgG (Vector Laboratories, Burlingame, CA, USA). Unbound Ig were removed by washing, and slides were incubated with NeutrAvidin Alkaline Phosphatase Conjugated (Thermo Scientific Inc., Waltham, MA, USA).

IHC on formalin-fixed, paraffin-embedded samples was performed with the following primary Abs: anti-CD3 (Dako, Glostrup, DK), anti-B220 (RA3–6B2, BD Pharmingen, San Jose, CA), anti-Foxp3 (FJK-16s; eBioscience, San Diego, CA), anti-GR-1 (RB6–8C5; BioLegend, San Diego, CA), anti-LAP TGF-β (R&D Systems, Minneapolis, MN), anti- and anti-IL6 (Santa Cruz Biotechnology, CA, USA), anti-TNFα (52B83; Abcam, Cambridge, UK) Abs. Sections were treated with H_2_O_2_/3% for 5 min to inhibit endogenous peroxidase and then washed in H_2_O. Antigen was unmasked with heat-induced epitope retrieval in ethylenediaminetetracetic acid (EDTA) buffer at pH 9 prior to incubation with anti-CD3, anti-GR-1, and anti-TNFα Abs and in sodium citrate buffer pH6 prior to incubation with anti-Foxp3, anti-LAP TGF-β and anti-IL6 Abs. The slices were then held for 20 min at room temperature. After washing in PBS/Tween-20, sections were incubated for 30 min with the primary Ab followed by incubation with the biotinylated goat anti-rabbit Ab (Vector Laboratories) to detect CD3, IFNγ and IL6, biotinylated rabbit anti-rat to detect GR1, Foxp3 and B220, biotinylated horse anti-goat to detect LAP TGF-β and biotinylated horse anti-mouse to detect TNFα. Then, sections were stained with Streptavidin Peroxidase (Thermo Scientific) followed by DAB as chromogen (Dako). The primary Ab was replaced with 10% non-immune serum for negative controls. Further controls were obtained by omitting the secondary Ab. Controls were always negative.

For CD11b/GR-1, double staining on formalin-fixed paraffin-embedded samples, sections were deparaffinized, subjected to antigen retrieval, treated with H_2_O_2_/3% for 5 min to inhibit endogenous peroxidase, and then washed in H_2_O. The slices were then incubated for 30 min with the first primary antibody CD11b (EPR1344; Abcam, Cambridge, UK) followed by incubation with the biotinylated goat anti-rabbit Ab and by detection with Streptavidin Peroxidase and DAB as chromogen.

Then, sections were incubated for 30 min with the second primary antibody GR-1 followed by incubation with the biotinylated rabbit anti-rat Ab and by detection with NeutrAvidin Alkaline Phosphatase Conjugated and Fast Red (Biocare Medical, Concord, CA) as chromogen.

### 8-oxoG determinations

8-OxoG was measured by HPLC/EC as previously described [19]. Following DNA extraction, RNase treatment and enzymatic hydrolysis, DNA hydrolysate was analysed by HPLC/EC (Coulochem I, Esa, Chelmsford, MA, USA) using a C18 250646 mm 5 mm Uptishere column (Interchim, San Pedro, CA, USA) equipped with a C18 guard column. The eluent was 50 mM ammonium acetate, pH 5.5, containing 9% methanol, at a flow rate of 0.7 ml/min. Deoxyguanosine was measured in the same run of corresponding 8-oxodG with a UV detector (model SPD-2A; Shimadzu, Kyoto, Japan) at 256 nm.

### Analysis of leucocytes population

To obtain a single cell suspension, spleen was gently disaggregated with a pestle and then with a syringe in 1 ml PBS1x, 2 mM EDTA, after letting debris decant for few minutes, the superior phase was transferred in a new tube. The following procedure was applied to both spleen single cell suspensions and blood samples: after erythrocyte depletion with ACK lysis buffer, 10^6^ cells were suspended in 100 μl RPMI, 10 mM Hepes, 1 mM EDTA, 2% FBS and labeled for 30′ at RT with 1 μg of the following antibodies: CD4-APC (eBioscience, SanDiego, CA, USA), CD8-PeCy7 (eBioscience), CD11b-FITC (eBioscience), B220-PacificBlue (BioLegend, San Diego, CA, USA), Ly-6G-PE (BioLegend), Ly-6C-APCeFluor780 (eBioscience). After labeling cells were washed twice with PBS1x, suspended in 200 μl PBS1x 2 mM EDTA and analyzed with BD LSR II Flow Cytometer (BD Biosciences, San Jose, CA, USA).

### Analysis of cytokines expression

Mice colon, previously minced and frozen, was suspended in 2 ml homogenization buffer (20 mM Tris-HCl pH 7.5, 2 mM EDTA, 2 mM EGTA, 250 mM sucrose, 5 mM DTT, 1 mM PMSF, 10 mM NaF and protease inhibitor) and homogenized with Turrax Homogenizer (IKA, Staufen, Germany). Samples were then sonicated and passed through a 1 ml syringe a few times. After adding TritonX-100 to a final concentration of 0, 1%, samples were mixed on rotating wheel for 30 min at +4°C. Protein extracts were then purified through centrifugation (300 g, 15′, +4°C) and supernatants were stocked at −80°C. Cytokines were analyzed with custom Bio-Plex Pro™ Mouse Cytokine 8-plex Assay (BioRad, Hercules, CA, USA) using the Bio-Plex MagPix system (BioRad) with Software Manager 6.1, according to manufacturer's instructions.

### Statistical analyses

Fisher's exact test was used to compare differences in the number and frequency of each histotype of colonic adenomas. Differences in immune cell counts between tumors from *Mutyh*^−/−^ and WT mice were assessed by Student's *t* test. Data are mean ± standard-deviation (SD). The SPSS software, version 11.0 (IBM, Armonk, NY, USA), was employed, with *P* < 0.05 as the significance cut-off.

## SUPPLEMENTARY FIGURES


