# Supplementary material for: The MUTYH base excision repair gene protects against inflammation-associated colorectal carcinogenesis
Source: Oncotarget. 2015 Jun 18;6(23):19671–84. doi: 10.18632/oncotarget.4284 (PMC4637313; doi:10.18632/oncotarget.4284)
Supplement: Supplementary file 1 [file oncotarget-06-19671-s001.pdf]

SUPPLEMENTARY FIGURES

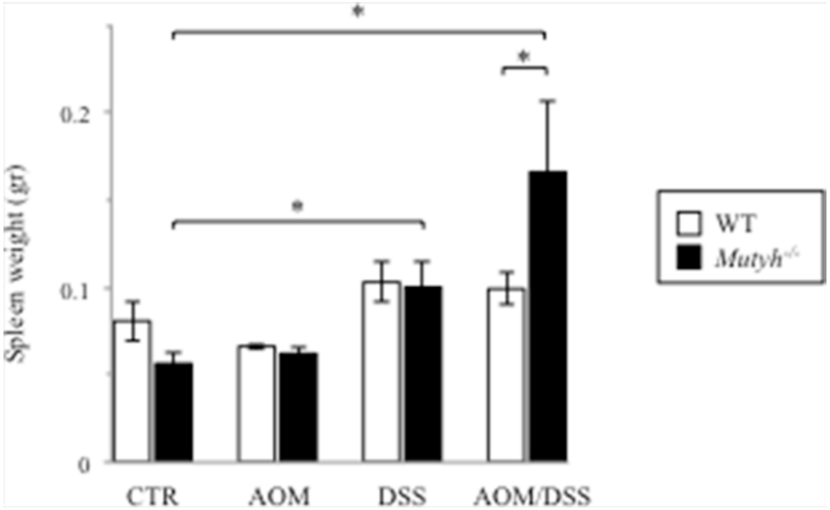

Supplementary Figure S1: Spleen weights following single and combined AOM and DSS exposures. Spleen weights were measured at the end of the treatment (80 days) in untreated (control, CTR) or AOM-, DSS- or AOM/DSS-treated mice. Wild-type (open bar) and *Mutyh*$^{-/-}$ (full bar) mice. Data are mean ± SE of 5–10 animals/genotype. *$p \leq 0.05$ (Student's *t*-test)

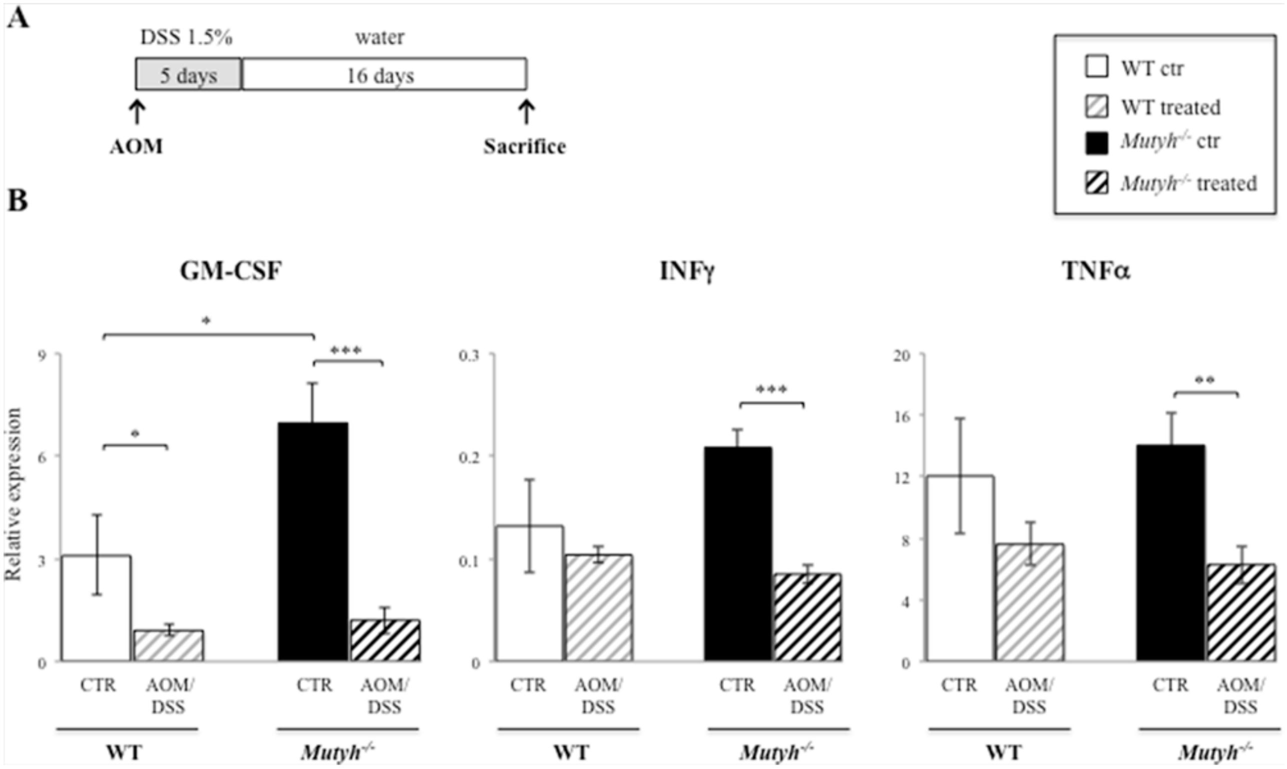

Supplementary Figure S2: Analysis of cytokines expression in the colon. **A.** Schematic representation of the treatment. **B.** Cytokines levels in the colon of untreated and treated wide-type mice (open and grey dashed bar, respectively) and untreated and treated *Mutyh*$^{-/-}$ mice (full and black dashed bar) were measured by the Bio-Plex Pro™ Mouse Cytokine 8-plex Assay. Proteins levels are expressed as concentration (pg/ml)/mg of total proteins. Data are mean ± SE of 4–5 animals/genotype. *$p \leq 0.05$ (Student's *t*-test).
